# Balancing speed and experience: the cognitive and affective impacts of playback acceleration in digital media consumption

**DOI:** 10.3389/fpsyg.2025.1664580

**Published:** 2025-11-20

**Authors:** Rich Yueh, Jonathan Lim, Ye Li, Rasam Dorri

**Affiliations:** School of Business, University of California, Riverside, Riverside, CA, United States

**Keywords:** playback speed, video acceleration, cognitive engagement, media design, speaker perception, user satisfaction, time-compressed media, educational technology

## Abstract

Playback-speed controls are ubiquitous on media platforms, yet their effects on viewers’ experience and outcomes are not fully understood. We examined how acceleration influences cognitive engagement, affective experience (satisfaction, discomfort, perceived distortions), speaker perceptions, and behavioral intentions, and whether motivational cues (quiz expectancy) shape these effects. Three preregistered experiments exposed adults to either procedural or conceptual videos at standard or accelerated speeds. Studies 2a–2b additionally manipulated quiz expectancy and included a brief objective quiz. Outcomes were assessed with multi-item self-report scales; Study 2a–2b quiz scores provided objective performance. Analyses used ANOVA with study-specific factors. Across studies, faster playback reliably increased perceived distortions and modestly reduced satisfaction. Effects on cognitive engagement, discomfort, and speaker favorability were limited and context-dependent (varying by content type and audience). Quiz expectancy sometimes enhanced engagement at faster speeds, particularly among students. Behavioral intentions to reengage with content showed no reliable differences by speed, and objective quiz performance did not differ by speed. Playback speed is a consequential design variable whose effects depend on content demands, audience characteristics, and motivational context. Thoughtful, context-aware integration of speed controls—especially in educational settings—can help preserve experiential quality without sacrificing efficiency.

## 1 Introduction

The global rise of digital media consumption has transformed how users interact with audiovisual content. A growing number of platforms such as YouTube, TikTok, Netflix, and Coursera allow viewers to adjust playback speed, typically ranging from 0.25x to 2.0x, as a way to save time and align content delivery with personal preferences (Lang et al., 2020; Murphy et al., 2023). Originally implemented to enhance accessibility and self-paced learning (Perifanou and Economides, 2022), playback speed controls have evolved into a widely adopted strategy across domains (Tran et al., 2024). As accelerated viewing becomes more common, it is essential to understand the cognitive, affective, and behavioral consequences of this practice within diverse media contexts.

Playback speed may appeal to users for multiple reasons, including matching personal attention spans, skipping redundant segments, or increasing productivity during educational consumption (Lang et al., 2020). This flexibility supports individualized learning and time management goals, yet introduces trade-offs between speed, comprehension, and user experience quality. Despite its prevalence, psychological research on playback speed remains limited, with most investigations situated within educational frameworks that prioritize learning performance (Cheng et al., 2022; Mo et al., 2022). Such a narrow focus overlooks broader experiential factors such as cognitive engagement, satisfaction, perceived speaker credibility, and willingness to reengage with content. Addressing this gap, the present study frames playback speed as a media psychology phenomenon that influences cognitive effort, affective evaluation, and behavioral intention in a digitally accelerated world. By applying media psychology principles, researchers can better understand the trade-offs users make between efficiency and engagement in contexts ranging from educational tutorials to entertainment and news.

This work draws on Cognitive Load Theory (Sweller et al., 2011), agenda-based regulation (Ariel et al., 2009), and paralinguistic communication frameworks (Lee et al., 2021; Smith and Shaffer, 1995) to explain how playback speed may shape user experience. Faster playback can reduce pauses and streamline delivery, potentially lowering extraneous cognitive load (Murphy et al., 2023). However, it may also overwhelm working memory when content is dense or unfamiliar, amplifying intrinsic cognitive load and reducing comprehension (Mo et al., 2022; Nagahama and Morita, 2017). User expectations further shape these outcomes: when viewers anticipate being tested, they may allocate more cognitive resources to process accelerated content effectively (Ariel et al., 2009; Murphy et al., 2022). Beyond cognitive effort, playback speed can disrupt perceptual fluency (Alter and Oppenheimer, 2009), increasing discomfort (Tran et al., 2024) and perceived distortions in audio or visual quality (Ritzhaupt et al., 2008), which can undermine satisfaction (Ritzhaupt et al., 2015) and social judgments of speaker credibility (Schwarz et al., 2021).

To investigate these dynamics, this research examines how playback speed affects four primary domains: cognitive engagement, affective and perceptual experiences (including satisfaction, discomfort, and distortions), speaker perception, and behavioral intentions. We also explore whether motivational cues—specifically quiz expectations—moderate these effects. Across three experiments involving both procedural and conceptual video content, we test how accelerated delivery interacts with user expectations and content demands to shape overall experience. By situating playback speed as a meaningful design variable, this study aims to inform more thoughtful platform design, educational strategy, and user awareness in an era of increasingly self-paced media consumption.

## 2 Methods

To evaluate the effects of video playback speed on viewer’s experiences, speaker perceptions, and behavioral intentions, we conduct three experimental studies manipulating video speed. To improve generalizability, we used two different videos—one involving procedural learning and one involving more conceptual learning—that differed in terms of their intrinsic cognitive load.

Specifically, procedural learning denotes the acquisition of an ordered sequence of actions that reliably produces a specific outcome (e.g., executing the steps of a tiramisu recipe). By contrast, conceptual abstraction involves constructing a relational understanding among ideas (e.g., currency value, purchasing power parity, and the Big Mac Index) that supports generalization and transfer to novel cases. Within the cognitive-load framework, conceptual learning carries higher intrinsic load due to the need for abstract schema construction; therefore, increases in playback speed are more likely to increase this load further than would a procedural learning video relying on step-by-step execution.

Study 1 serves as the foundational test of speed effects for a cooking tutorial video with procedural learning content. Study 2a uses a video with more conceptual content about an economic concept and adds a quiz manipulation to test the role of motivational framing. Study 2b replicates and extends these effects within a student population to assess consistency across age and context.

### 2.1 Participants

We recruited participants who were 18 years of age or older and had internet access. For Study 1 and Study 2a, we recruited participants through Lucid Theorem (now called Cint: https://www.cint.com/products/theorem/), an online platform that provides access to nationally representative samples. For Study 2b, we recruited students from a large public university in the western United States who completed the study in exchange for course credit.

The gender distribution was relatively balanced across studies, with a slightly higher proportion of female participants in Study 1 (55.5%) and Study 2a (53.0%), and a slightly higher proportion of male participants in Study 2b (52.8%). The average age was 47.92 years (*SD* = 16.66) in Study 1, 47.90 years (*SD* = 16.42) in Study 2a, and 24.74 years (*SD* = 9.71) in Study 2b. Additional demographic details are provided in the Appendix.

### 2.2 Measures

#### 2.2.1 Video watching experiences

To assess video-watching experiences, we developed a questionnaire to gauge participants’ perceptions of their affective and cognitive experience when watching the video. Items included: “I found this video entertaining,” “I was quite focused on the video,” and “The video playback seemed fast.” See Appendix for full wording of all items. Participants responded to each item on a 5-point Likert scale from 1 (*strongly disagree*) to 5 (*strongly agree*). Principal axis factor (PAF) analysis revealed four factors: (1) viewer satisfaction (6 items, Study 1 *α* = 0.91; Study 2a α = 0.88; Study 2b α = 0.87), (2) viewer discomfort (3 items, Study 1 α = 0.78; Study 2a α = 0.77; Study 2b α = 0.70), (3) cognitive engagement (3 items, Study 1 α = 0.78; Study 2a α = 0.74; Study 2b α = 0.75), and (4) perceived distortions (3 items, Study 1 α = 0.72; Study 2a α = 0.80; Study 2b α = 0.74) (see Appendix for factor loadings by study). The perceived distortions factor assessed participants’ subjective impressions of playback quality and speed, including alterations in auditory characteristics, visual presentation, and perceived playback speed. Items were reverse-scored where necessary so that higher scores reflected greater levels of the respective factor. Mean scores were calculated for each factor.

#### 2.2.2 Speaker perceptions

We asked participants to “Please rate your agreement with the following statements about the speaker in the video” on 18 descriptors developed to measure six theoretically-derived dimensions: confidence, intelligence, skill, warmth, passion, and authenticity. Participants responded on a 5-point Likert scale ranging from 1 (*strongly disagree*) to 5 (*strongly agree*). Following a PAF analysis, the scale was reduced to 12 items that loaded on a single factor. The six excluded items loaded on a secondary factor that consisted exclusively of negatively-worded statements. This pattern suggests that participants may have experienced confusion or response bias when interpreting negatively-phrased items, resulting in an artifactual factor. The retained items demonstrated a coherent single-factor structure across all three studies, reflecting a clear and consistent underlying construct. The 12 items were then averaged to form a composite score representing overall speaker perceptions with higher scores reflecting more positive perceptions. Internal consistency was high across all studies (Study 1 *α* = 0.96; Study 2a α = 0.95; Study 2b α = 0.91).

#### 2.2.3 Behavioral intentions

Behavioral intentions were assessed using four items designed to measure participants’ likelihood to engage further with the content: “try out the recipe” in Study 1 and “tell anyone about what you learned in this video” in Studies 2a and 2b, along with “share this video with friends and/or family,” “watch more videos from this YouTube channel,” and “subscribe to this YouTube channel.” Responses were recorded on a 5-point scale ranging from 1 (*definitely not*) to 5 (*definitely yes*). Reliability was strong across studies (Study 1 α = 0.93; Study 2a α = 0.92; Study 2b α = 0.83). A mean composite score was calculated, with higher values indicating greater intention to behaviorally engage further.

#### 2.2.4 Quiz for studies 2a and 2b

We designed a 5-item multiple choice quiz for Studies 2a and 2b to assess objective understanding and learning. Each item had a single correct response, and a total score ranging from 0 to 5 was computed, with higher scores indicating greater learning. The quiz evaluated participants’ understanding of the economic principles and factual details presented in the video, including the theoretical foundation of the Big Mac Index, its global relevance, the approximate number of countries in which Big Macs are sold, representative pricing information, and familiarity with related indices mentioned in the material. Example items included “The Big Mac Index is based on…” (correct answer: Purchasing Power Parity Theory) and “Why have Big Macs generally been accepted as a reliable index?” (correct answer: Big Macs are sold almost everywhere).

### 2.3 Procedure

Participants completed all studies online via Qualtrics. After providing informed consent, participants were instructed to watch the assigned video without interruptions before answering questions about the video and their experience watching it.

Study 1 used a between-subjects design in which participants were randomly assigned to view a tiramisu cooking tutorial (https://www.youtube.com/watch?v=uNowLq9fm9I) at either 1x, 1.25x, or 1.5x speed. We selected this cooking tutorial as an example of procedural learning content. The video required viewers to follow a clear sequence of observable actions to achieve a concrete outcome, representing a task with low conceptual abstraction and correspondingly low intrinsic cognitive load. Its content was designed to elicit minimal conceptual reasoning demands and moderate cognitive engagement typical of step-based, hands-on instructional material.

In Studies 2a and 2b, participants watched an educational video about the Big Mac Index (https://www.youtube.com/watch?v=b71uzrr8hUs) at either 1x or 1.25x speed. We selected this video as an example of conceptual learning content. It introduced more abstract concepts such as currency value and purchasing power parity and was designed to impose moderate intrinsic load and encourage sustained cognitive engagement.

Studies 2a and 2b used a 2 (playback speed: 1x vs. 1.25x) × 2 (motivational framing: informed vs. not informed) factorial design to examine whether motivational framing—operationalized as forewarning participants about an upcoming quiz—would moderate the effects of video speed. Participants in the quiz-informed condition were told they would be asked questions about the video, although all participants completed the same quiz and post-video measures.

At the end of each study, participants were provided with a link to the video they watched, allowing them to review the content if so desired.

### 2.4 Pre-registration and data quality

All three studies were pre-registered on AsPredicted.org prior to data collection (Study 1: https://aspredicted.org/9yt4-dwjx.pdf; Study 2a: https://aspredicted.org/6qcn-39zk.pdf; Study 2b: https://aspredicted.org/86bw-pxg4.pdf).

We safeguarded data quality by using the following pre-registered exclusion criteria: if participants (a) completed the experiment in a time less than three standard deviations below the mean log completion time, (b) straight-lined their responses (i.e., provided identical or nearly identical answers across questions on the same rating scale; Kim et al., 2018), (c) completed less than 90% of the survey, or (d) did not self-report honest responses. Self-reported honesty was operationalized with a single item administered at the end of the survey: “I responded to this survey honestly,” with response options of “yes,” “mostly yes,” “mostly no,” and “no.” Only participants who selected “yes” were retained for the analyses. No additional attention checks were included in the survey. In addition, English proficiency was assessed with a single self-report item: “Please rate your level of English proficiency,” with response options of “beginner,” “conversational,” “business,” “fluent,” and “native.” We did not exclude any participants on language ability as they all met the level of “business” or higher. Study 1 recruited 694 participants (final sample size *N* = 326; 53.0% excluded), Study 2a recruited 686 participants (final sample size *N* = 313; 54.4% excluded), and Study 2b recruited 329 participants (final sample size *N* = 246; 25.2% excluded). We report recruitment and exclusion rates for transparency. Analyses were conducted on the sample after applying the pre-registered data quality screens. We acknowledge these sizable exclusion rates as a limitation; the results should be interpreted as applying to participants who met our high data quality standards.

Certain deviations from the original analytical plans should be noted. Although speaker perception was originally pre-registered as six separate outcome variables, analyses in the present manuscript used a single composite score due to high inter-item correlations, in order to reduce Type I error and improve interpretability. Similarly, the 15 “Other DVs” dependent variables were named in the preregistrations individually, but we will be analyzing them in four factors—specifically cognitive engagement, discomfort, satisfaction, and perceived distortions. In Studies 2a and 2b, motivational framing was part of the experimental design but was not pre-registered as a formal moderator; as a result, interaction analyses involving this variable are also considered exploratory. We also report the pre-registered mediation results in Appendix due to the complexity of the results. Finally, Study 2a was pre-registered with a target sample size of 400 participants, but the final dataset included 313 usable responses after applying exclusion criteria.

## 3 Results

### 3.1 Study 1: baseline test of playback speed effects on viewer experience

**Figure 1:**
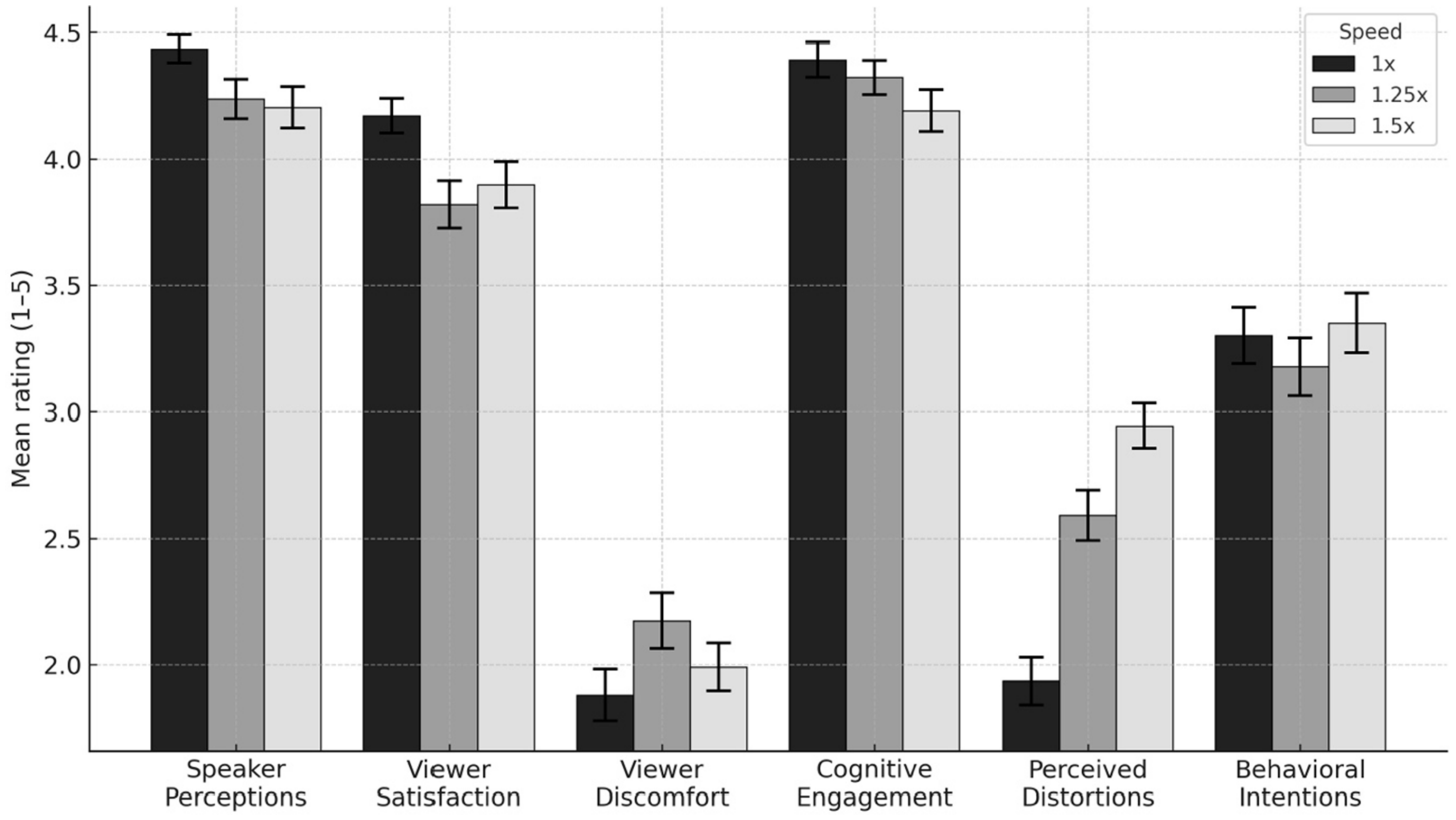
Descriptive statistics by speed for Study 1.

To evaluate the impact of playback speed (1x, 1.25x, 1.5x) on viewer experience for the cooking tutorial video, we conducted one-way analyses of variance (ANOVAs) to examine the effect of playback speed on each outcome. Descriptive statistics and ANOVA results for all outcome variables are provided in Table 1, while Figure 1 provides a visual summary of the descriptive data.

**Table 1:** Means, standard deviations, and ANOVA results for viewer experience and speaker perceptions for Study 1.

Outcome variable	1x (*M, SD*)	1.25x (*M, SD*)	1.5x (*M, SD*)	*F*	*p*	$\eta_{p}^{2}$
Speaker perceptions	4.44 (0.58)	4.24 (0.79)	4.20 (0.88)	2.93	0.055	0.018
Viewer satisfaction	4.17 (0.72)	3.82 (0.95)	3.90 (0.98)	4.57	0.011	0.027
Viewer discomfort	1.88 (1.07)	2.17 (1.12)	1.99 (1.02)	2.05	0.13	0.013
Cognitive engagement	4.39 (0.71)	4.32 (0.69)	4.19 (0.89)	1.96	0.14	0.012
Perceived distortions	1.94 (0.98)	2.59 (1.01)	2.94 (0.94)	30.30	<0.001	0.160
Behavioral intentions	3.30 (1.16)	3.18 (1.16)	3.35 (1.26)	0.59	0.55	0.004

Playback speed significantly influenced several aspects of both viewer experience and perceptions of the speaker. The effect of playback speed on participants’ overall perceptions of the speaker was marginally significant (*p* = 0.055), with speakers being rated higher at 1x speed (*M* = 4.44, *SD* = 0.58) than at 1.25x (*M* = 4.24, *SD* = 0.79) and 1.5x speeds (*M* = 4.20, *SD* = 0.88). Playback speed also significantly impacted viewer satisfaction (*p* = 0.011), with viewers experiencing higher satisfaction when watching the video at 1x speed (*M* = 4.17, *SD* = 0.72) than at 1.25x (*M* = 3.82, *SD* = 0.95) and 1.5x speeds (*M* = 3.90, *SD* = 0.98). Playback speed also significantly impacted perceived distortions in the video (*p* < 0.001). Ratings of distortion increased with speed, with participants in the 1x condition reporting the least distortion (*M* = 1.94, *SD* = 0.98), followed by those in the 1.25x (*M* = 2.59, *SD* = 1.01), and 1.5x conditions (*M* = 2.94, *SD* = 0.94). Notably, these distortion ratings were still significantly lower than the scale maximum of five.

Playback speed did not have a statistically significant effect on other outcomes. For viewer discomfort, the effect of speed was nonsignificant but suggestive (*p* = 0.13): Discomfort was highest at 1.25x (*M* = 2.17, *SD* = 1.12), compared to 1.5x (*M* = 1.99, *SD* = 1.02) and 1x (*M* = 1.88, *SD* = 1.07). Similarly, the effect of speed on cognitive engagement was nonsignificant but suggestive (*p* = 0.14), with the highest ratings for 1x (*M* = 4.39, *SD* = 0.71) compared to 1.25x (*M* = 4.32, *SD* = 0.69) and 1.5x (*M* = 4.19, *SD* = 0.89). Finally, behavioral intentions did not significantly vary across playback speeds (*p* = 0.55).

#### 3.1.1 Discussion

Study 1 found divergent effects of faster speeds on experience and cognition for a procedural learning video. Relative to 1x speed, faster playback lowered satisfaction, slightly reduced speaker favorability (marginal), and increased perceived audiovisual distortions, while self-reported cognitive engagement and behavioral intent remained largely unchanged. This pattern is consistent with the idea that faster playback adds extraneous load by making the signal feel less natural (for example, choppier audio or visual flow), which degrades the viewing experience and, in turn, perceptions of the speaker, without necessarily reducing viewers’ stated effort or behavioral intent.

Practically, this points to a modest time–experience trade-off for procedural learning videos: viewers can save time when watching at faster speeds, but at some cost to how the content and speaker are received. Based on these results, we should expect equal or stronger penalties when the video requires an even higher level of conceptual abstraction, as this would place even greater cognitive demand on viewers. Study 2a tests this shift by using more conceptually-demanding content and introduces motivational framing (i.e., forewarning a quiz) to examine whether a light motivational cue can offset the experiential costs of speed.

### 3.2 Study 2a: do effects replicate for a more conceptual, abstract video, and do quiz expectations moderate the effects of speed?

Study 2a examined whether the impact of playback speed on viewer experience could be moderated by motivational framing. Specifically, we tested whether informing participants about an upcoming quiz would change the way they engaged with a cognitively-demanding video viewed at normal or increased speed. The study used a 2 (speed: 1x vs. 1.25x) × 2 (motivational framing: informed vs. not informed about quiz) between-subjects design. The video used in this study—a brief economics explainer—was selected to introduce higher cognitive demand than the procedural video used in Study 1.

**Figure 2:**
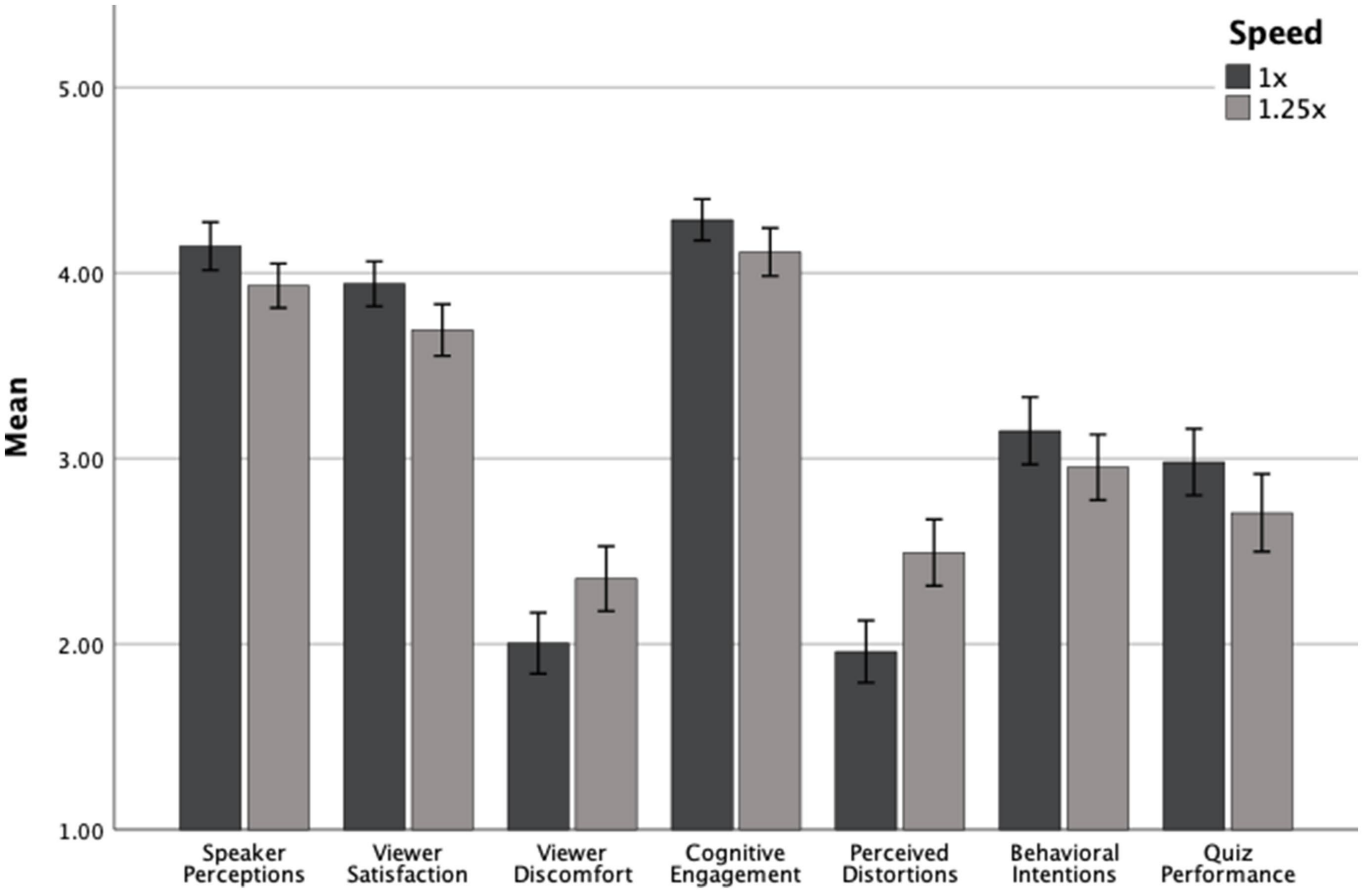
Dependent variables by speed for Study 2a, collapsed across quiz informed condition.

Table 2 and Figure 2 summarize the descriptive statistics for Study 2b and Table 3 shows the results of ANOVA analyses. Consistent with the results of Study 1, participants in the 1x condition rated the speaker more positively than those in the 1.25x condition (*M* = 4.14, *SD* = 0.82 vs. *M* = 3.93, *SD* = 0.76; *p* = 0.022), had higher viewer satisfaction (*M* = 3.94, *SD* = 0.77 vs. *M* = 3.69, *SD* = 0.88; *p* = 0.007), and reported less distortion (*M* = 1.96, *SD* = 1.06 vs. *M* = 2.49, *SD* = 1.14; *p* < 0.001) and viewer discomfort (*M* = 2.00, *SD* = 1.04 vs. *M* = 2.35, *SD* = 1.11; *p* = 0.005). Playback speed did not have a statistically significant effect on behavioral intentions (*p* = 0.11). Unlike in Study 1, participants also reported greater cognitive engagement at 1x (*M* = 4.29, *SD* = 0.71) than at 1.25x (*M* = 4.11, *SD* = 0.82; *p* = 0.045). Finally, participants in the 1x condition scored somewhat higher on the 5-item quiz than those in the 1.25x condition (*M* = 2.98, *SD* = 1.13 vs. *M* = 2.71, *SD* = 1.33; *p* = 0.086), but this effect was only marginally significant.

**Table 2:** Descriptive analysis by speed and quiz condition for Study 2a.

Speed	Quiz informed	Speaker perceptions	Viewer satisfaction	Viewer discomfort	Cognitive engagement	Perceived distortions	Behavioral intentions	Quiz performance
		*Mean (SD)*						
1x	No	4.22 (0.74)	3.89 (0.77)	2.00 (1.03)	4.28 (0.73)	1.88 (1.03)	3.07 (0.11)	3.08 (1.14)
	Yes	4.05 (0.90)	4.00 (0.77)	2.01 (1.06)	4.30 (0.69)	2.05 (1.10)	3.24 (1.19)	2.86 (1.12)
	Total	4.14 (0.82)	3.94 (0.77)	2.00 (1.04)	4.29 (0.71)	1.96 (1.06)	3.15 (1.15)	2.98 (1.13)
1.25x	No	3.88 (0.87)	3.72 (0.92)	2.32 (1.06)	4.13 (0.83)	2.45 (1.13)	3.05 (1.03)	2.60 (1.30)
	Yes	3.98 (0.63)	3.66 (0.85)	2.38 (1.16)	4.10 (0.82)	2.54 (1.15)	2.85 (1.20)	2.81 (1.35)
	Total	3.93 (0.76)	3.69 (0.88)	2.35 (1.11)	4.11 (0.82)	2.49 (1.14)	2.95 (1.12)	2.71 (1.33)
Total	No	4.06 (0.82)	3.81 (0.85)	2.15 (1.05)	4.21 (0.78)	2.15 (1.11)	3.06 (1.07)	2.85 (1.24)
	Yes	4.01 (0.77)	3.82 (0.83)	2.21 (1.13)	4.19 (0.76)	2.31 (1.15)	3.04 (1.21)	2.83 (1.24)
	Total	4.04 (0.79)	3.82 (0.84)	2.18 (1.09)	4.20 (0.77)	2.23 (1.13)	3.05 (1.14)	2.84 (1.24)

**Table 3:** ANOVA analysis of playback speed and quiz informed condition effects on dependent variables for Study 2a.

Viewer outcomes	Speed (1x vs. 1.25x)			Quiz Informed (yes or no)			Speed x Quiz Informed		
	*F*	*p*	$\eta_{p}^{2}$	*F*	*p*	$\eta_{p}^{2}$	*F*	*p*	$\eta_{p}^{2}$
Speaker perceptions	5.28	0.022	0.017	0.14	0.71	<0.001	2.24	0.14	0.007
Viewer satisfaction	7.33	0.007	0.023	0.074	0.79	<0.001	0.74	0.39	0.002
Viewer discomfort	8.09	0.005	0.026	0.089	0.77	<0.001	0.049	0.83	<0.001
Cognitive engagement	4.04	0.045	0.013	0.001	0.97	<0.001	0.10	0.75	<0.001
Perceived distortions	17.68	<0.001	0.054	1.05	0.31	0.003	0.11	0.74	<0.001
Behavioral intentions	2.54	0.11	0.008	0.017	0.90	<0.001	2.11	0.15	0.007
Quiz performance	3.54	0.061	0.011	0.004	0.95	<0.001	2.39	0.12	0.008

Contrary to expectations, the motivational framing manipulation did not significantly impact any outcome. There were no significant main effects or interaction effects of prior knowledge about the quiz on speaker perception, satisfaction, discomfort, cognitive engagement, perceived distortions, or behavioral intentions (all *ps* > 0.05), indicating that the motivational framing did not alter how speed influenced viewer responses.

#### 3.2.1 Discussion

For conceptual learning videos, Study 2a largely replicated the effects of faster playback as found in Study 1—relative to 1x speed, 1.25x reduced satisfaction and increased perceived audiovisual distortions—and extends it by revealing a drop in reported cognitive engagement at 1.25x that was not evident for the procedural learning video used in Study 1. In other words, when intrinsic cognitive demands are higher, adding extra speed-related load may have negative cognitive effects, not just affective ones.

Motivational framing did not change outcomes in this sample; neither main effects nor interactions with speed emerged. One interpretation of this null result is that such cues are context dependent: for a participant sample in which quizzes are not especially valued, the prompt neither buffers the experiential costs of acceleration nor increases engagement. This potential boundary condition motivates Study 2b’s focus on students, for whom accountability cues are routine and may more strongly calibrate how viewers regulate effort and speed.

### 3.3 Study 2b: replication in a student sample

Study 2b replicated the design of Study 2a using a sample of undergraduate students at a large public university. This enabled us to test the generalizability of playback speed and motivational framing effects in a younger, more academically-oriented population that may be more used to watching faster video content. Participants were randomly assigned to one of four conditions in a 2 (speed: 1x vs. 1.25x) × 2 (motivational framing: informed vs. not informed about quiz) between-subjects design, viewing the same economics explainer video used in Study 2a.

**Figure 3:**
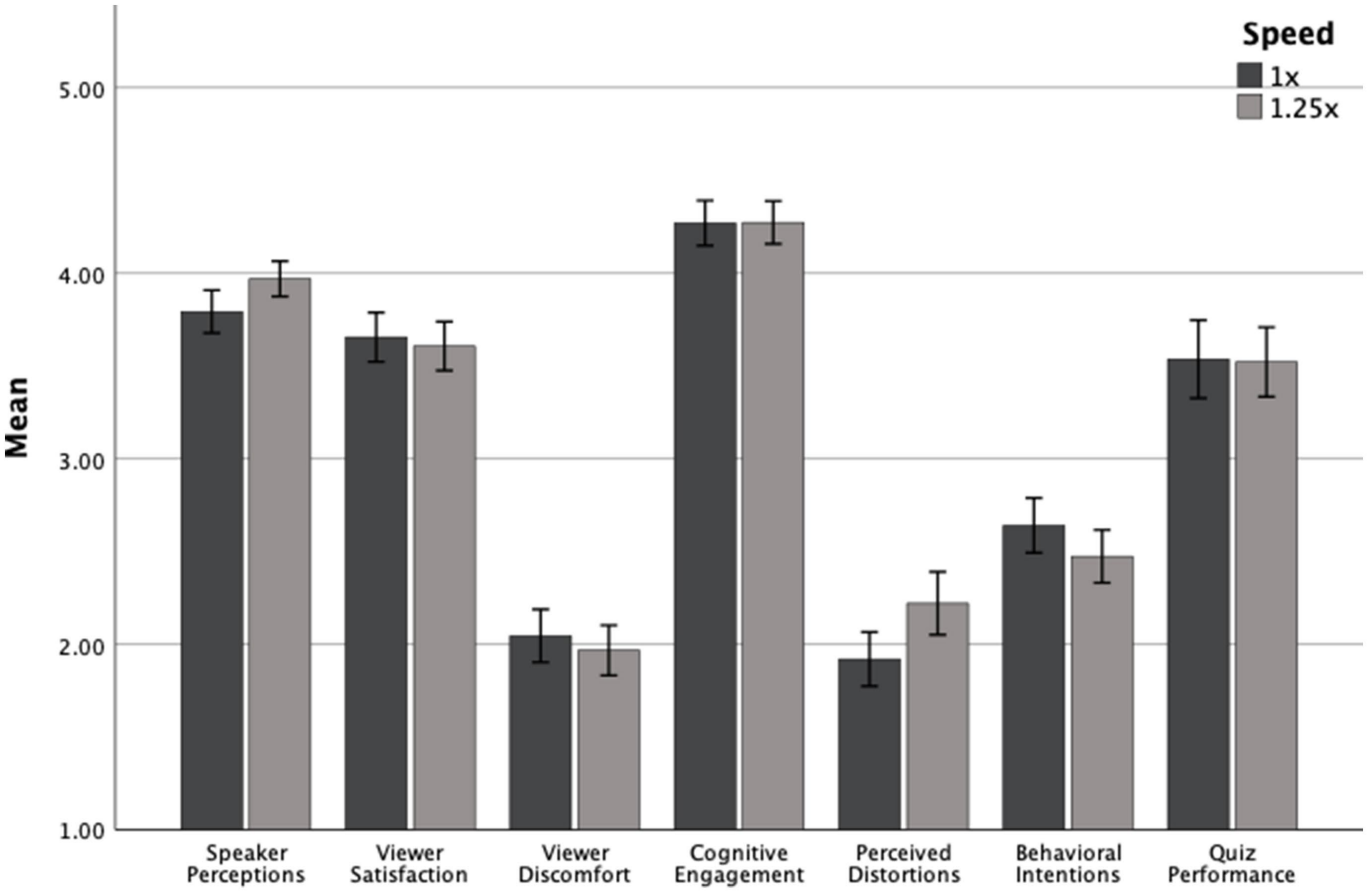
Dependent variables by speed for Study 2b, collapsed across quiz informed condition.

Table 4 and Figure 3 summarize the descriptive statistics for Study 2b. As seen in the results of ANOVA analyses depicted in Table 5, playback speed significantly influenced participants’ evaluations of the speaker and their perceptions of video distortion. Specifically, participants in the 1.25x condition rated the speaker more positively (*M* = 3.97, *SD* = 0.53) than those in the 1x condition (*M* = 3.79, *SD* = 0.65). Playback speed also significantly influenced perceived distortion: participants in the 1.25x condition reported more distortion (*M* = 2.22, *SD* = 0.94) than those in the 1x condition (*M* = 1.92, *SD* = 0.82). No other main effects of speed were statistically significant. Viewer satisfaction, discomfort, cognitive engagement, and behavioral intent did not differ meaningfully by playback speed (all *ps* > 0.05). Finally, participants in the 1x condition scored similarly on the 5-item quiz as those in the 1.25x condition (*M* = 3.52, *SD* = 1.04 vs. *M* = 3.54, *SD* = 1.19; *p* = 0.99).

**Table 4:** Descriptive analysis by speed and quiz condition for Study 2b.

Speed	Quiz informed	Speaker perceptions	Viewer satisfaction	Viewer discomfort	Cognitive engagement	Perceived distortions	Behavioral intentions	Quiz performance
		*Mean (SD)*						
1x	No	3.72 (0.61)	3.56 (0.82)	2.13 (0.82)	4.39 (0.65)	1.95 (0.83)	2.54 (0.89)	3.45 (1.24)
	Yes	3.87 (0.69)	3.75 (0.67)	1.96 (0.79)	4.15 (0.70)	1.89 (0.82)	2.74 (0.77)	3.62 (1.14)
	Total	3.79 (0.65)	3.65 (0.75)	2.04 (0.81)	4.27 (0.69)	1.92 (0.82)	2.64 (0.83)	3.54 (1.19)
1.25x	No	4.02 (0.52)	3.45 (0.72)	2.01 (0.76)	4.14 (0.65)	2.31 (0.96)	2.36 (0.80)	3.36 (1.23)
	Yes	3.92 (0.54)	3.76 (0.72)	1.92 (0.75)	4.41 (0.60)	2.13 (0.92)	2.59 (0.77)	3.68 (0.79)
	Total	3.97 (0.53)	3.61 (0.73)	1.97 (0.75)	4.27 (0.64)	2.22 (0.94)	2.47 (0.79)	3.52 (1.04)
Total	No	3.87 (0.58)	3.51 (0.77)	2.07 (0.79)	4.26 (0.66)	2.12 (0.91)	2.45 (0.85)	3.41 (1.23)
	Yes	3.89 (0.62)	3.75 (0.69)	1.94 (0.77)	4.28 (0.67)	2.01 (0.88)	2.66 (0.77)	3.65 (0.98)
	Total	3.88 (0.60)	3.63 (0.74)	2.01 (0.78)	4.27 (0.66)	2.07 (0.89)	2.56 (0.82)	3.53 (1.12)

**Table 5:** ANOVA analysis of playback speed and quiz condition effects on dependent variables for Study 2b.

Viewer outcomes	Speed (1x vs. 1.25x)			Quiz informed (yes or no)			Speed x quiz informed		
	*F*	*p*	$\eta_{p}^{2}$	*F*	*p*	$\eta_{p}^{2}$	*F*	*p*	$\eta_{p}^{2}$
Speaker perceptions	5.46	0.020	0.022	0.11	0.75	<0.001	2.62	0.11	0.011
Viewer satisfaction	0.25	0.62	0.001	7.06	0.008	0.028	0.47	0.49	0.002
Viewer discomfort	0.64	0.43	0.003	1.64	0.20	0.007	0.15	0.70	0.001
Cognitive engagement	0.002	0.97	<0.001	0.060	0.81	<0.001	9.26	0.003	0.037
Perceived distortions	7.05	0.008	0.028	0.99	0.32	0.004	0.29	0.59	<0.001
Behavioral intentions	2.56	0.11	0.010	4.23	0.041	0.017	0.020	0.89	<0.001
Quiz performance	0.009	0.93	<0.001	2.97	0.086	0.012	0.30	0.59	0.001

In contrast to Study 2a, motivational framing exerted a stronger influence on certain viewer responses. Participants who were informed about an upcoming quiz reported significantly higher satisfaction (*M* = 3.75, *SD* = 0.69) than those who were not informed (*M* = 3.51, *SD* = 0.77). Similarly, quiz-informed participants reported stronger behavioral intent (*M* = 2.66, *SD* = 0.77) than those in the uninformed condition (*M* = 2.45, *SD* = 0.85). No other main effects of motivational framing were significant. Speaker evaluations, discomfort, cognitive engagement, and perceived distortions did not significantly vary by motivational framing (*ps* > 0.05).

A significant interaction between playback speed and motivational framing was found for cognitive engagement, *F* = 9.26, *p* = 0.003, ηp^2^ = 0.037 (see Table 5). Among participants who viewed the video at 1.25x speed, those who were informed about the quiz reported higher engagement (*M* = 4.41, *SD* = 0.60) than those who were not (*M* = 4.14, *SD* = 0.65). In contrast, for participants in the 1x condition, those who were not informed about the quiz reported greater engagement (*M* = 4.39, *SD* = 0.65) than those who were informed (*M* = 4.15, *SD* = 0.70). This crossover pattern suggests that motivational framing may enhance engagement at faster speeds but may slightly suppress it at a standard speed. No other interaction effects were significant (*ps* > 0.05).

#### 3.3.1 Discussion

Overall, participants in Study 2b exhibited more positive speaker perceptions in the faster speed condition, along with no effects on their satisfaction, discomfort, or engagement, a contrast from the results seen in Study 2a. This could be due to the differences in sampling, as university students tend to be more familiar with digital learning environments and accustomed to using increased speeds (Perifanou and Economides, 2022).

Additionally, the interaction between speed and motivational framing might be best explained through the lens of CLT (Sweller et al., 2011). When learners expected a quiz, they likely understood that they would need to allocate greater cognitive energy to schema construction and deeper processing, and this mental “budgeting” was enough to cover the greater cognitive load required for moderate acceleration (1.25x speed). In contrast, participants not expecting a quiz may have engaged less at the 1.25x speed because they did not actively regulate their cognitive resources in anticipation of an assessment, instead processing the video at a more passive level. These results align with research on test expectancy and cognitive regulation (Ariel et al., 2009), which suggests that learners strategically adjust their cognitive effort based on anticipated task demands.

## 4 General discussion

We aimed to examine the effect of video playback speed on video-watching experiences, speaker perception, and behavioral intentions in an instructional setting. Three experiments explored both the direct effects of playback speed and the moderating role of motivational framing on how learners engage with instructional videos. Overall, we found that playback speed significantly influenced several dimensions of the video-watching experience but had limited effects on speaker perceptions and behavioral intentions. These findings extend prior research that focused narrowly on learning performance by showing that even modest changes in playback speed can shape satisfaction, engagement, and social impressions. By situating playback speed as a media design variable, this study highlights the need for more thoughtful integration of speed controls with attention to content demands and audience expectations. Below, we interpret our findings in light of existing research, address their limitations, and consider their implications for theory, practice, and future investigation.

### 4.1 Video-watching experiences

For viewer satisfaction, we found that participants reported lower satisfaction at increased playback speeds in Study 1 and Study 2a. This finding is consistent with past research showing that accelerated playback speeds reduce enjoyment (Ritzhaupt et al., 2015; Tran et al., 2024). In addition, across all studies, perceived distortions increased with playback speed, corroborating prior findings that unnatural pacing affects the viewing experience (Ritzhaupt et al., 2008). Viewer discomfort, however, was only significantly impacted in Study 2a, where participants reported greater discomfort in the 1.25x condition compared to the 1x condition.

A potential explanation pertains to differences in participant expectations regarding cognitive effort and video pacing. In Study 1, which used a nationally representative sample, participants watched a recipe video, a procedural task that does not require sustained cognitive effort. Even if certain participants were unaccustomed to increased speeds, the flexibility of the content likely made acceleration less disruptive, resulting in no significant discomfort. In Study 2a, participants viewed an economics lecture, a type of content that may have already placed substantial cognitive load on them. When the video was sped up to 1.25x, this added further load, thus creating a sense of difficulty or unease that in turn led to greater discomfort. As mentioned previously, the absence of increased discomfort at 1.25x speed in Study 2b may be attributed to differences in the participant sample. University students’ greater comfortability with digital learning environments may have made them less likely to perceive 1.25x speed as unnatural or cognitively demanding, thus reducing discomfort.

Playback speed consistently influenced subjective impressions across studies, particularly speaker perception, satisfaction, and perceived distortions. These findings suggest that even modest speed increases may reduce perceived video quality and enjoyment, while more cognitive or behavioral outcomes remain relatively stable. Regarding cognitive engagement, Study 1 did not reveal a significant effect of playback speed, whereas Study 2a showed that participants at 1x speed reported higher engagement than those at 1.25x speed. Furthermore, Study 2b identified a significant interaction effect between quiz expectations and playback speed, indicating that the effect of playback speed on engagement depended on whether participants expected to be tested after viewing the video. Specifically, learners who were informed about the quiz in advance showed greater engagement at 1.25x speed than those at 1x speed, potentially due to their mental “budgeting” being able to account for the greater cognitive load needed for moderate acceleration (1.25x speed). Taken together, these results reinforce the notion that playback speed has a robust influence on how cognitively-demanding video content is experienced, even in the presence of motivational cues. However, they also suggest that a simple quiz prompt may not be sufficient to mitigate the perceptual or experiential drawbacks associated with increased speed.

### 4.2 Speaker perceptions

Regarding speaker perceptions, Study 1 found an effect of playback speed, with participants rating the speaker more favorably at 1x speed than at 1.25x or 1.5x. Study 2a confirmed this pattern, showing a statistically significant preference for the speaker in the 1x condition over the 1.25x condition. In contrast, Study 2b revealed a reversed effect: participants rated the speaker more favorably at 1.25x than at 1x. These opposing patterns suggest that the impact of playback speed on speaker perception may be moderated by participant characteristics such as age or familiarity with digital learning environments. The findings of Study 2b align with prior research on speech rate and credibility (Smith and Shaffer, 1995), which suggests that moderate acceleration can enhance perceptions of confidence and competence. However, in contrast to studies indicating that excessively fast speech rates reduce warmth and trustworthiness (Smith et al., 1975), the current research did not find a uniform negative effect of increased playback speed on speaker favorability, likely due to differences across participant samples.

Specifically, Studies 1 and 2a participants were recruited from a nationally representative sample, which likely included individuals with varying levels of exposure to sped-up video content. If participants were less accustomed to accelerated playback, they may have perceived the 1.25x speed as unnatural or difficult to follow, contributing to lower speaker favorability ratings. Conversely, Study 2b recruited students from a large public university, a group that may be more familiar with sped-up lecture videos commonly used in online education (Perifanou and Economides, 2022). As a result, the student participants may have been more receptive to the faster speech rate, interpreting it as a sign of confidence and efficiency, leading to higher speaker favorability ratings at 1.25x speed. These mixed effects across studies suggest that perceptions of speaker credibility are not uniformly affected by faster playback, but instead may depend on the viewer’s prior experience, expectations, and comfort with accelerated speech. While some users may interpret faster speech as a sign of competence, others may experience it as rushed or unnatural, based on their level of familiarity with sped-up content. This underscores the need to consider audience characteristics when evaluating how presentation speed influences social impressions.

### 4.3 Behavioral intentions

Across all three studies, playback speed did not significantly influence participants’ behavioral intentions – that is, their likelihood of engaging further with the content by sharing the video, subscribing to the channel, or trying the featured recipe. This suggests that while playback speed can shape viewers’ subjective impressions and experiences, it may not meaningfully impact downstream behaviors. One possible explanation is that behavioral intent is more strongly driven by perceived interest, content relevance, or personal learning goals than by presentation speed.

However, Study 2b revealed a significant main effect of motivational framing on behavioral intentions: participants who were informed about the upcoming quiz reported greater intent to engage with the content than those who were not. This suggests that motivational cues, such as test expectancy, can enhance behavioral engagement, particularly among students who are accustomed to academic evaluation contexts. In contrast, this effect did not emerge in Study 2a’s more diverse, general population sample, indicating that the impact of such prompts may be shaped by contextual or demographic factors.

Overall, while playback speed influenced subjective impressions, it did not meaningfully alter behavioral intentions on its own. Thus, the differences between Study 2a and Study 2b – particularly in the influence of motivational framing – highlight the importance of audience characteristics. Whereas quiz prompts did not influence outcomes among general population adults, they did significantly increase satisfaction and behavioral intentions among university students. This suggests that motivational framing may be more effective in contexts where test expectancy is normative or where users are primed to regulate effort in response to task demands. These findings emphasize that design strategies targeting cognitive engagement or behavioral response should consider not only the content and delivery but also the demographic and contextual factors that shape user expectations and regulation strategies.

### 4.4 Limitations

Despite the contributions of the current research, certain limitations should be acknowledged. First, the range of playback speeds examined was limited to moderate increases (1.25x and 1.5x), preventing a thorough exploration of potential non-linear effects at higher acceleration levels. Prior research suggests that comprehension remains stable up to 2.0x speed but deteriorates beyond that threshold (Murphy et al., 2022). By restricting playback speed manipulation to 1.5x in Study 1 and 1.25x in Studies 2a and 2b, the study may have not adequately captured the full range of cognitive and perceptual changes that might emerge at more extreme speeds. This limitation is particularly relevant given that many learners engage with online educational content at speeds exceeding 1.5x, raising questions about the generalizability of these findings to real-world viewing behaviors. If playback speed effects are not linear, the trends observed in this study may not extrapolate to more extreme accelerations, making it unclear whether comprehension and engagement declines gradually or whether a critical threshold exists beyond which video-based learning becomes ineffective.

An additional limitation concerns the study-specific instrument used to assess video-watching experiences. The 15-item questionnaire was developed specifically for this study in order to better understand participants’ viewing experience. While demonstrating adequate internal consistency across all three studies, it has not been validated through independent psychometric evaluation. Future research should subject the questionnaire to rigorous validation procedures or rely on already validated measurement scales for robustness and generalizability.

Furthermore, content type varied across studies, introducing a confounding factor that complicates the interpretation of playback speed effects. Study 1 used a recipe video, while Studies 2a and 2b featured economics lectures, each of which imposes different kinds of cognitive demand on learners. Given that procedural and conceptual learning rely on distinct cognitive processes, it is possible that the impact of playback speed varies depending on the nature of the material. Differences in content structure may thus influence participants’ cognitive load and engagement independently of playback speed, confounding the effects observed in this study. Without controlling for these differences, it remains uncertain whether the patterns identified here generalize across different learning contexts or whether they are specific to the particular content types used in this research.

Finally, the study relied on self-reported measures for key dependent variables, such as cognitive engagement, satisfaction, and behavioral intentions, which introduce the potential for bias. While self-reports provide insight into learners’ subjective experiences, they are susceptible to social desirability effects, recall distortions, and individual differences in self-perception accuracy (Podsakoff et al., 2003). For instance, participants may have overestimated their engagement levels due to demand characteristics or underestimated their discomfort to align with perceived expectations about the benefits of faster playback. In addition, self-reports fail to capture real-time cognitive fluctuations that occur during video viewing, limiting the ability to assess moment-to-moment engagement and attentional shifts. As a result, the study’s conclusions about engagement and satisfaction should be interpreted with caution, as they reflect participants’ perceptions rather than direct measures of cognitive effort or behavioral outcomes.

### 4.5 Implications

The widespread availability of playback speed controls across digital platforms demands a clearer understanding of how these tools shape not just momentary comprehension, but broader patterns of user experience and attention regulation. This research stresses the need to treat playback speed not merely as a user convenience feature, but as an active design element that influences media processing at multiple levels.

One key implication concerns how playback speed is integrated into platform architecture. Current designs typically default to standard or accelerated speeds without providing users with meaningful guidance about how different settings may affect emotional engagement, speaker perception, or long-term viewing habits. To improve user experience, platforms should consider implementing context-aware recommendations, for example, adjusting suggested speeds based on content type (e.g., explanatory, emotional, narrative-driven) or user engagement goals (e.g., learning vs. passive viewing). Also, platforms could experiment with user feedback indicators, such as previewing audio changes at different speeds, or offering short prompts about potential effects on processing fluency, satisfaction, or perception of the speaker.

There are also implications for content creators, who may not realize that their delivery style, emotional tone, and perceived credibility can be distorted at higher playback speeds, even though the change is externally imposed by the user or platform. When creators produce emotionally rich or persuasive content (e.g., personal narratives, advocacy messages), they may need to anticipate how accelerated pacing affects the reception of their tone and presence. Content may benefit from built-in tempo buffers, such as natural pauses, to remain comprehensible and authentic across different playback rates.

More broadly, the normalization of accelerated playback may have unintended consequences for attentional development, particularly among younger users. Increasing exposure to time-compressed media could train users to expect faster information flow, reducing tolerance for slower, more reflective content. Over time, this may contribute to diminished attentional stamina, increased distractibility, and a preference for constant stimulation, patterns that closely resemble the attentional dysregulation associated with ADHD (Thorell et al., 2022). While the relationship between media tempo and ADHD has not been conclusively established, platforms and policymakers should consider how interface features that promote speed-first habits might interact with cognitive development and mental health over time.

As seen in our findings with student viewers, motivational prompts may enhance satisfaction and engagement in contexts where test expectancy is normative. This underscores the need to align platform cues with user context—especially in educational or high-effort settings—so that motivational scaffolds can effectively prime deeper involvement. Our findings suggest a need for media literacy interventions that help users understand not only what playback speed does, but what it means. Educational efforts could focus on helping users recognize the trade-offs between speed and emotional or social resonance, encouraging more deliberate, goal-aligned tempo choices. Rather than framing acceleration as a universal good, designers, educators, and users alike should consider playback speed as a tool that can either support or subvert communication quality, depending on how, when, and why it is used.

### 4.6 Future research

Although the present study provides a comprehensive account of how playback speed influences user experience, several key questions remain. One important direction for future research involves exploring the boundaries of user tolerance and adaptation to accelerated media. While this research focused on playback speeds up to 1.5x, real-world users often consume content at 2x or higher, particularly in educational contexts where efficiency is prioritized (Tharumalingam et al., 2025). It remains unclear whether the perceptual and experiential declines observed at 1.5x would intensify at more extreme speeds, or whether frequent users become desensitized and develop compensatory processing strategies. Further studies would be particularly valuable in this context, as they could assess patterns of habituation, learning outcomes, and cumulative cognitive load across a wider variety of speeds.

Additionally, the present study examined playback speed in single-session, controlled viewing conditions to allow for internal validity and experimental precision. However, naturalistic media consumption is highly dynamic, shaped by user goals, time constraints, content complexity, and environmental distractions. Real-world viewers often adjust playback speeds fluidly across and within sessions, using cues such as speaker clarity to regulate tempo. Future work should include within-subject, naturalistic field studies where viewers can choose their preferred speeds across sessions, thus better approximating real-world viewing situations. Experience sampling or ecological momentary assessment methods could help capture these adaptive behaviors in context and reveal how viewers navigate trade-offs between speed, fluency, and comprehension over time (Bolger and Laurenceau, 2013). These designs could also clarify whether the effects observed here generalize to mobile environments, multi-tasking contexts, or binge-watching patterns, where attention and emotional involvement may follow different trajectories.

Furthermore, individual differences likely play a key role in shaping how viewers experience and respond to time-compressed media. Traits such as need for cognition, habitual multitasking, or prior exposure to sped-up content may influence whether users find accelerated playback cognitively engaging or emotionally taxing. Some viewers may experience heightened efficiency and focus when content is delivered rapidly, while others may find that a faster pace undermines clarity, empathy, or retention. Identifying these individual difference moderators could help clarify for whom speed-based features are beneficial, neutral, or potentially counterproductive.

Finally, deeper psychophysiological and behavioral analyses could illuminate the mechanisms behind the observed effects. While the present studies relied on self-report measures of perception, engagement, and behavioral intent, future research could incorporate physiological and nonverbal indicators of attention and affect. Measures such as eye-tracking could provide insight into the attentional and emotional dynamics of time-compressed viewing (e.g., Guo and Hu, 2024). These techniques would also allow researchers to examine how playback speed influences perception at a nonconscious level, thus helping to further unpack how speed, context, and user characteristics interact to shape modern media experiences.

## 5 Conclusion

Taken together, these findings advance our understanding of how playback speed functions as an important design feature in the user experience. As digital platforms increasingly offer users control over the tempo of media consumption, playback speed has become a prevalent variable in shaping how people experience, evaluate, and respond to content. This study contributes to a more comprehensive understanding of playback speed’s influence, not only information processing, but also on affective response, cognitive perception, and behavioral engagement.

This research also raises critical questions about the cumulative effects of accelerated media exposure. While playback speed tools offer flexibility and productivity benefits, their widespread use may unintentionally shape user expectations for tempo, engagement, and attentional intensity in a variety of consequential ways. These concerns are particularly salient in light of emerging debates about digital media’s role in attentional erosion and the amplification of ADHD-like behaviors.

Ultimately, the psychological consequences of playback speed highlight the need for more intentional design, transparent user guidance, and public discourse about the trade-offs between speed, comprehension, and experiential quality. As media consumption continues to accelerate, the challenge will not be simply to keep up, but to ensure that speed does not come at the cost of connection, clarity, and cognitive integrity.

## Data Availability

The raw data supporting the conclusions of this article will be made available by the authors, without undue reservation.
