# Supplementary material for: Balancing speed and experience: the cognitive and affective impacts of playback acceleration in digital media consumption
Source: Front Psychol. 2025 Nov 20;16:1664580. doi: 10.3389/fpsyg.2025.1664580 (PMC12675162; doi:10.3389/fpsyg.2025.1664580)
Supplement: Supplementary file 1 [file Data_Sheet_1.docx]

# Appendix A: Study materials and PRINCIPAL Axis factor loadings

## Video experience items

Viewer satisfaction

1. I enjoyed this video
2. I found this video entertaining
3. I would like to watch other videos in this format
4. I found this video educational
5. I found this video relaxing
6. I found this to be a good use of my time

Viewer discomfort

1. Watching the video was a bad experience
2. I found this video stressful to watch
3. I felt busy after watching this video

Cognitive engagement

1. I was quite focused on the video
2. I fully understood the content of this video
3. I paid close attention to the video

Perceived distortions

1. The video playback seemed fast
2. The audio in the video seemed distorted or weird
3. The images in the video seemed distorted or weird

Response options: 1 = Strongly disagree; 2 = Somewhat disagree; 3 = Neither agree nor disagree; 4 = Somewhat agree; 5 = Strongly agree

## Speaker perception items (18 items presented in randomized order)

Authenticity

1. The speaker seems inauthentic. *(reverse coded)*
2. The speaker seems genuine.
3. The speaker seems trustworthy.

Passion

1. The speaker seems passionate about the subject matter.
2. The speaker seems to care about the subject matter.
3. The speaker seems unenthusiastic about the subject matter. *(reverse coded)*

Confidence

1. The speaker seems confident.
2. The speaker seems modest. *(reverse coded)*
3. The speaker seems assured.

Skill

1. The speaker seems knowledgeable about the subject matter.
2. The speaker seems to be an expert on the subject matter.
3. The speaker seems to be inexperienced in the subject matter. *(reverse coded)*

Intelligence

1. The speaker seems intelligent.
2. The speaker seems smart.
3. The speaker seems incapable. *(reverse coded)*

Warmth

1. The speaker seems warm.

17. The speaker seems likeable.

18. The speaker seems mean. *(reverse coded)*

Response options: 1 = Strongly disagree; 2 = Somewhat disagree; 3 = Neither agree nor disagree; 4 = Somewhat agree; 5 = Strongly agree

**Quiz items**

1. The Big Mac Index is based on:

- Economic Forecast Index
- Purchasing Power Parity Theory
- Hines-Schefter Hypothesis
- Big Mac Hypothesis
- I don't know

1. Why have Big Macs generally been accepted as a reliable index?

- McDonald’s paid for Big Macs to be featured
- Big Macs are sold almost everywhere
- Big Macs are one of the favorite food items around the world
- Big Macs are the same size in McDonald’s around the world
- I don't know

1. Big Macs are sold in about how many countries?

- I don’t know
- 120
- 130
- 140
- 150

1. A Big Mac in Poland costs approximately:

- $1.75
- $2.50
- $3.75
- $5.00
- I don’t know

1. Which of following is not an index mentioned in the video?

- Comparison Index
- Mini-Mac Index
- Billy Index
- All three were mentioned
- I don’t know

## PAF factor loadings for video experience DVs – Study 1

**
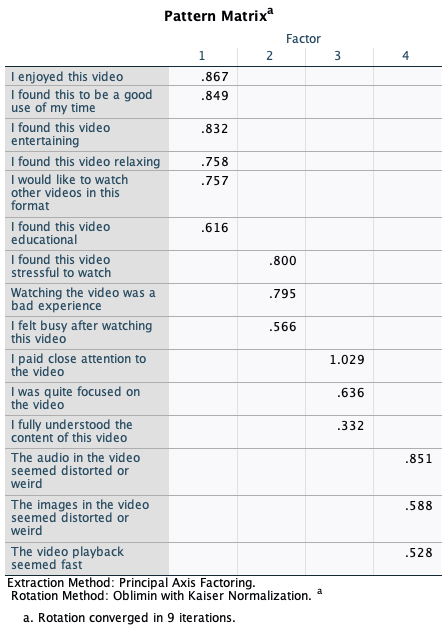
**

## PAF factor loadings for speaker perception items – Study 1

**
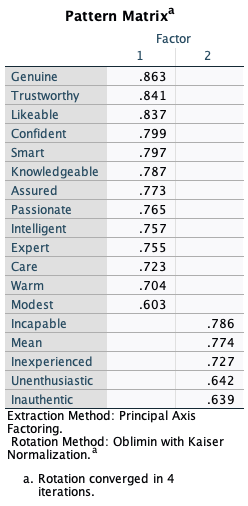
**

**
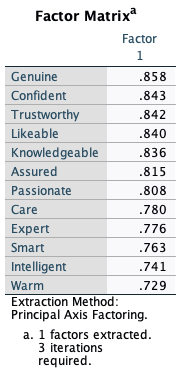
**

## PAF factor loadings for video experience DVs – Study 2a

***
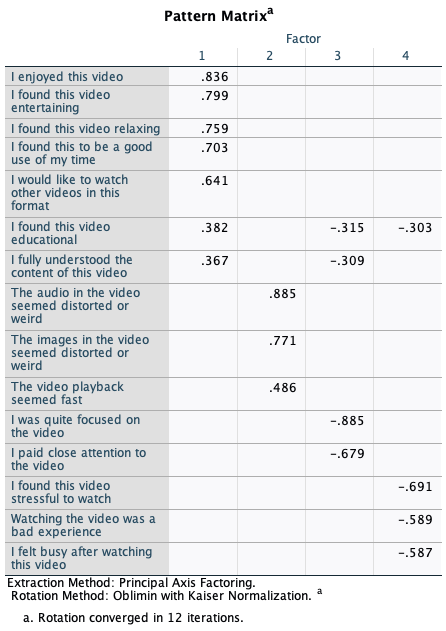
***

## PAF factor loadings for speaker perception items – Study 2a


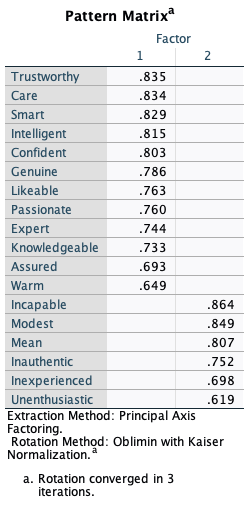


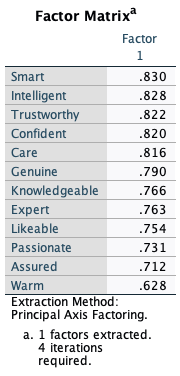


## PAF factor loadings for video experience DVs – Study 2b

***
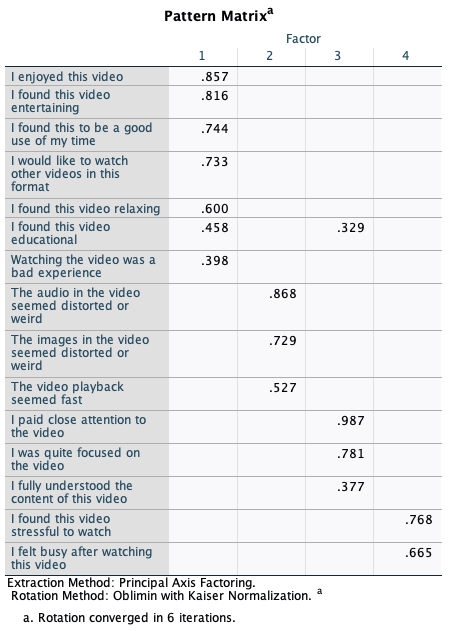
***

## PAF factor loadings for speaker perception items – Study 2b

**
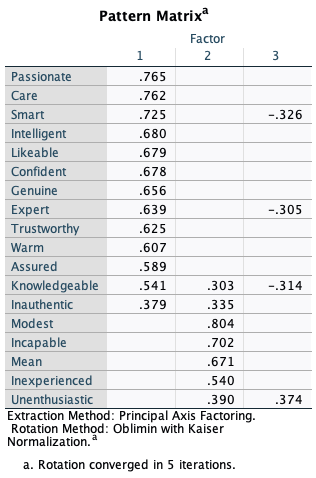
**

**
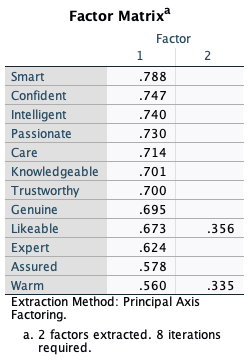
**

# Appendix B: demographic characteristics of study participants

|  | Study 1  (*N* = 326) | Study 2a  (*N* = 313) | Study 2b  (*N* = 246) |
| --- | --- | --- | --- |
| **Gender** |  |  |  |
| Male | 44.5 | 47.0 | 52.8 |
| Female | 55.5 | 53.0 | 47.2 |
| **Ethnicity** |  |  |  |
| White | 77.6 | 75.2 | 16.3 |
| Black or African American | 9.8 | 6.5 | 2.9 |
| Asian or Pacific Islander | 5.5 | 4.6 | 48.2 |
| American Indian or Alaska Native | 1.2 | 0.0 | 0.0 |
| Mixed race | 0.0 | 5.5 | 11.8 |
| Latino or Hispanic | 0.0 | 4.9 | 19.2 |
| Other | 3.7 | 3.3 | 1.6 |
| Prefer not to answer | 2.1 | 0.0 | 0.0 |
| **English Proficiency** |  |  |  |
| Business | 4.0 | 6.7 | 4.9 |
| Fluent | 19.0 | 17.3 | 32.1 |
| Native | 77.0 | 76.0 | 63.0 |
| **Age** *Mean (SD)* | 47.92 (16.66) | 47.90 (16.42) | 24.74 (9.71) |

# Appendix C. Does speaker perception mediate the relationship between playback speed and behavioral intentions?

As pre-registered in all three studies, we conducted mediation analysis using *PROCESS* Model 4 (Hayes, 2022) to examine if speaker perception significantly mediated the association between playback speed and behavioral intention (*N* = 326). The analyses employed 5,000 bootstrap resamples with HC3 heteroscedasticity-consistent standard errors.

For Study 1, playback speed was a significant negative predictor of speaker perception (path a: *b* = -0.12, *SE* = 0.05, *t* = -2.30, *p* = .022, *R*^2^ = .015), while speaker perception was a significant positive predictor of behavioral intention when controlling for playback speed (path b: *b* = 0.29, *SE* = 0.11, *t* = 2.73, *p* = .007, *R*^2^ = .035). The negative indirect effect of playback speed on behavioral intention via speaker perception was significant (path *ab*: *b* = -0.03, Boot*SE* = 0.02, 95% CI [-0.08, -0.003]. However, the total effect of playback speed on behavioral intention was not significant (path c: *b* = 0.03, *SE* = 0.08, *t* = 0.32, *p* = .75), nor was the direct effect when controlling for speaker perception (c′: *b* = 0.06, *SE* = 0.08, *t* = 0.73, *p* = .47). These results indicate that playback speed influenced behavioral intention indirectly through its effect on speaker perception, even though no total or direct effects of playback speed on behavioral intention were observed.

For Study 2a, playback speed was a significant negative predictor of speaker perception (path a: *b* = -0.21, *SE* = 0.09, *t* = -2.39, *p* = .018, *R*^2^ = .018), whereas speaker perception was a significant positive predictor of behavioral intention when controlling for playback speed (path b: *b* = 0.24, *SE* = 0.09, *t* = 2.59, *p* = .010; *R*^2^= .034). The indirect effect of playback speed on behavioral intention via speaker perception was negatively significant (path *ab*: *b* = -0.05, Boot*SE* = 0.03, 95% CI [-0.12, –0.004]). Nevertheless, the total effect of playback speed on behavioral intention was not significant (path c: *b* = -0.20, *SE* = 0.13, *t* = -1.53, *p* = .13), nor was the direct effect when controlling for speaker perception (path c′: *b* = –0.15, *SE* = 0.13, *t* = -1.13, *p* = .26). These findings indicate that playback speed influenced behavioral intention indirectly through its effect on speaker perception, in the absence of a significant total or direct effect.

Finally, for Study 2b, playback speed was a significant positive predictor of speaker perception (path a: *b* = 0.18, *SE* = 0.08, *t* = 2.33, *p* = .021; *R*^2^ = .022). Also, speaker perception was a significant positive predictor of behavioral intention when controlling for playback speed (path b: *b* = 0.44, *SE* = 0.09, *t* = 5.00, *p* < .001; *R*^2^ = .11). The positive indirect effect of playback speed on behavioral intention via speaker perception was significant (path *ab*: *b* = 0.08, Boot*SE* = 0.04, 95% CI [0.01, 0.15]). However, the total effect of playback speed on behavioral intention was not significant (path c: *b* = -0.17, *SE* = 0.10, *t* = -1.60, *p* = .11), whereas the direct effect when controlling for speaker perception was significantly negative (path c′: *b* = -0.25, *SE* = 0.01, *t* = –2.46, *p* = .015). Thus, playback speed exhibited a positive indirect association with behavioral intention through speaker perception, alongside a negative direct association with behavioral intention, yielding a non-significant total effect.
